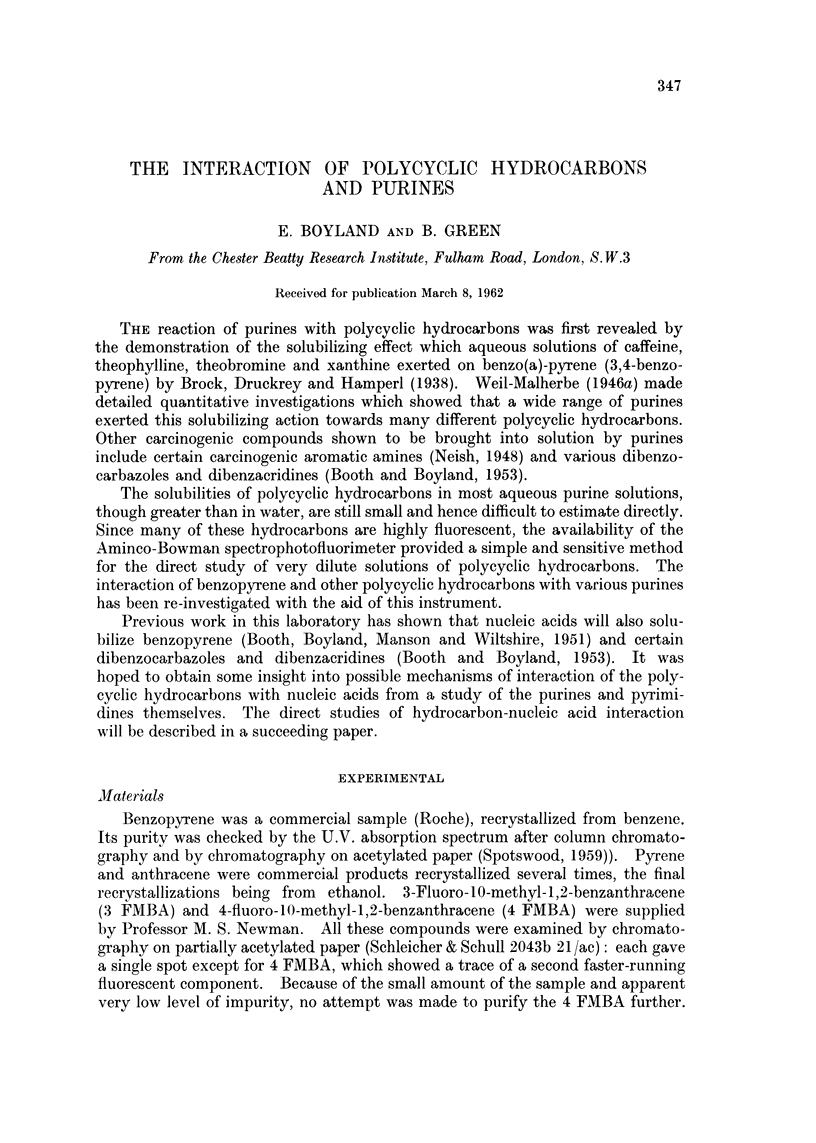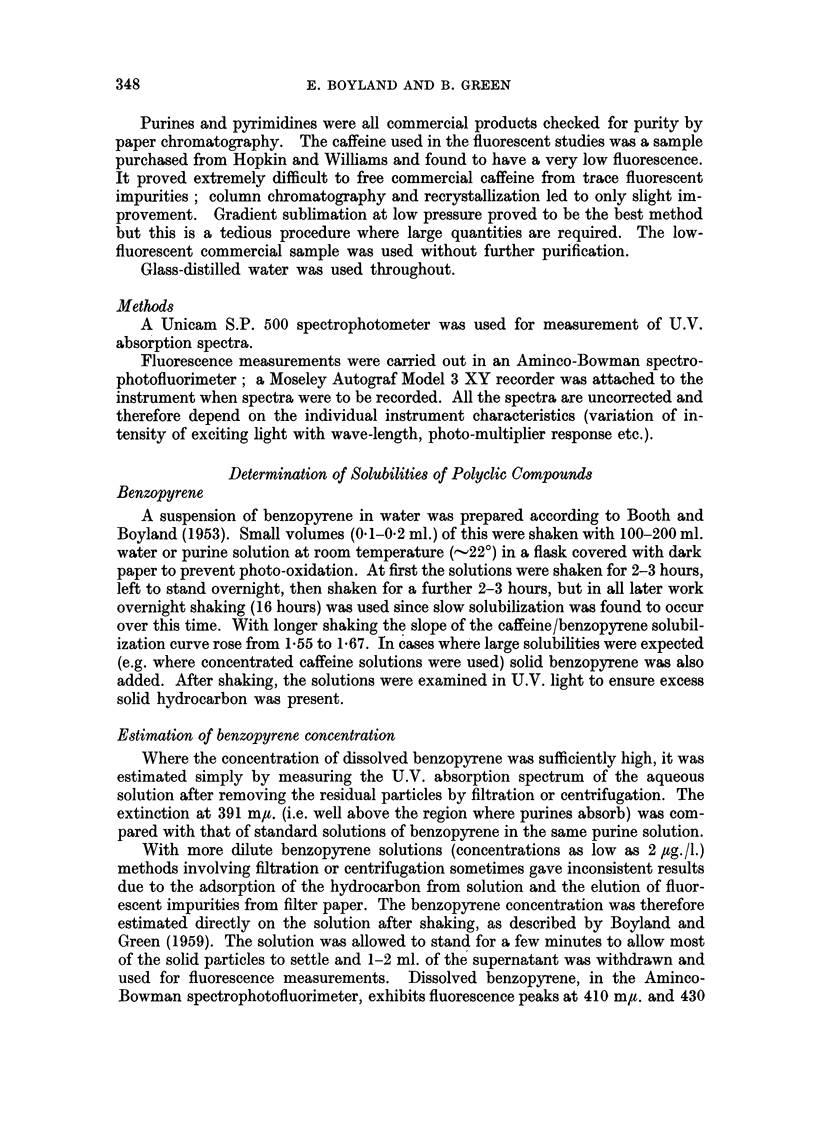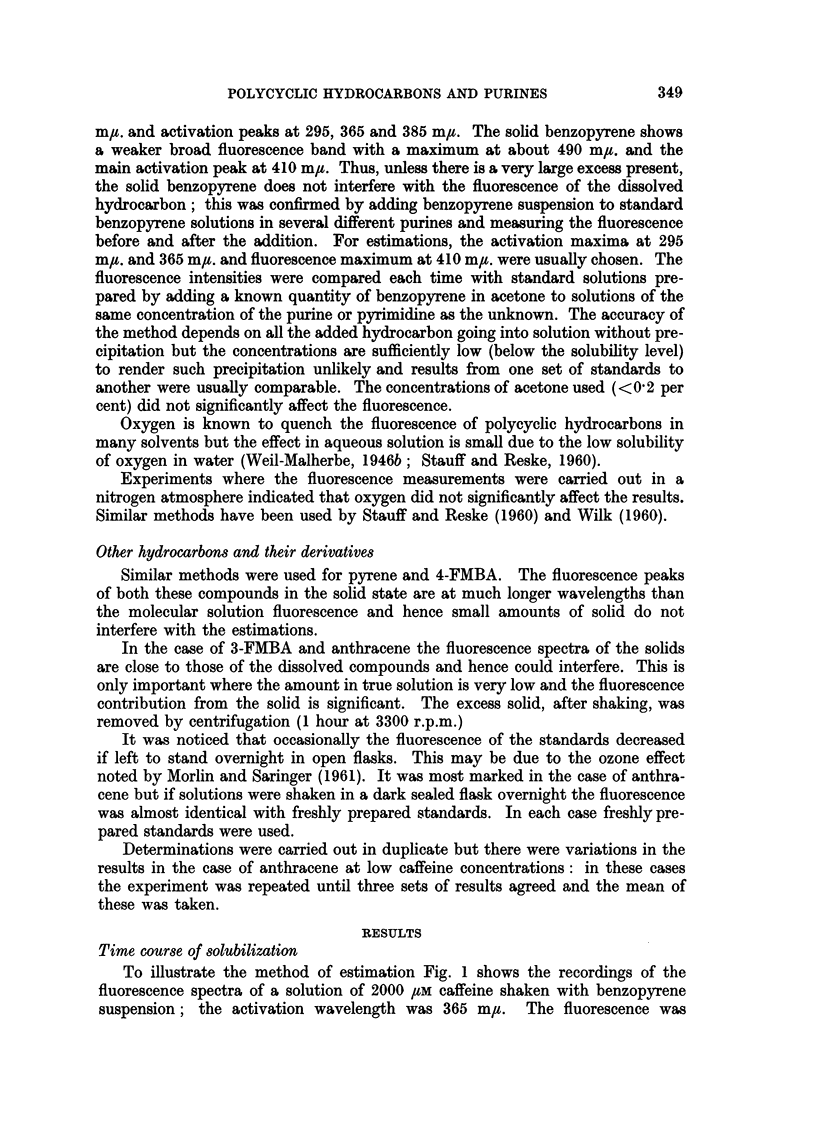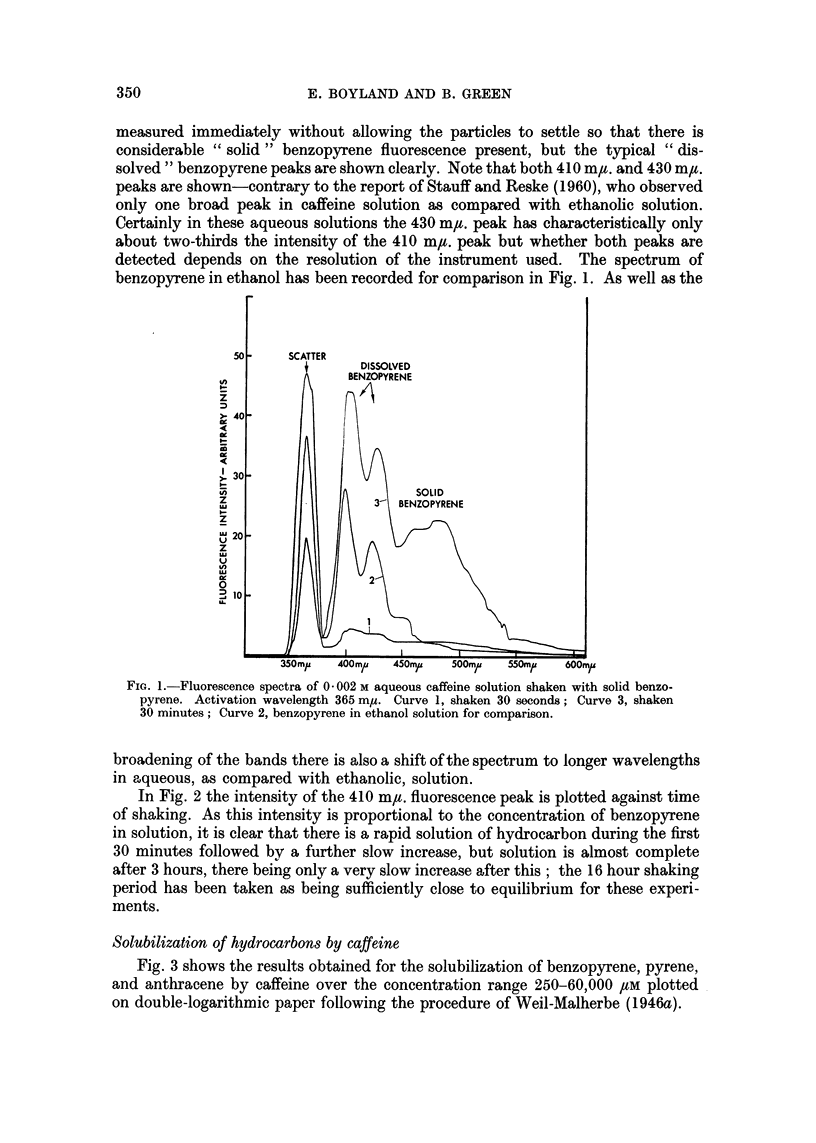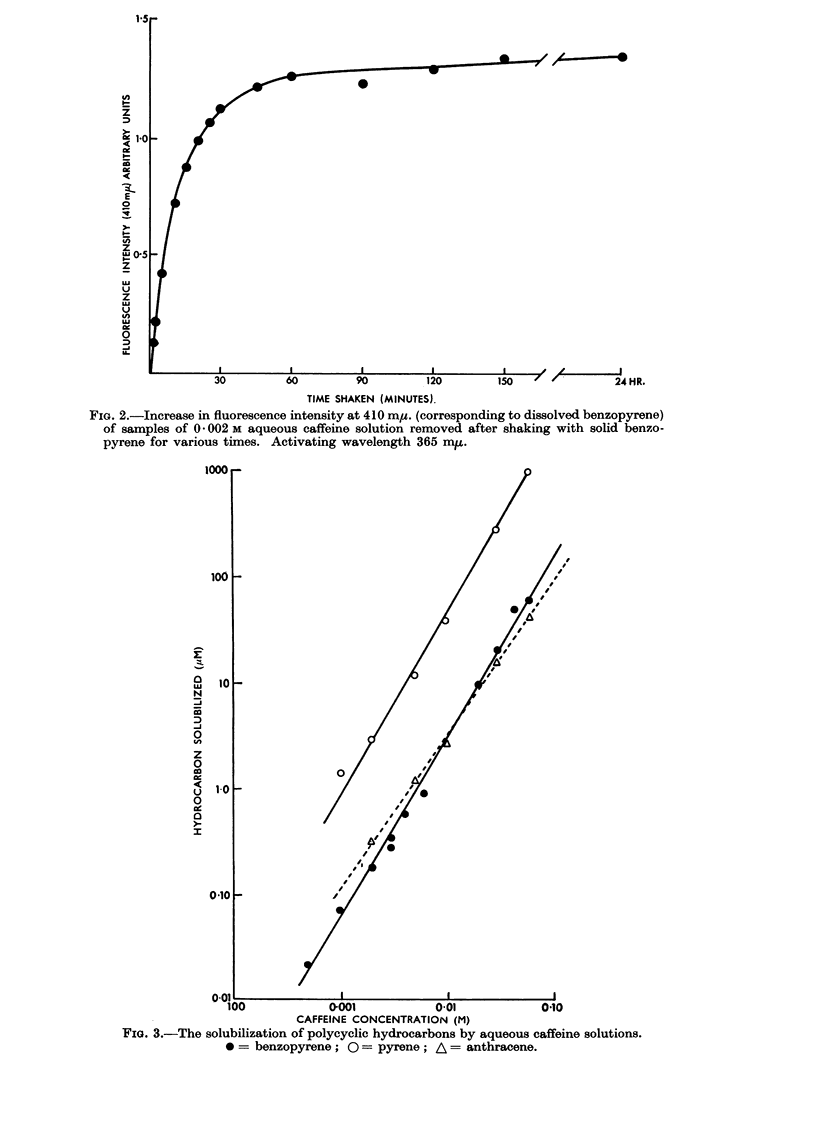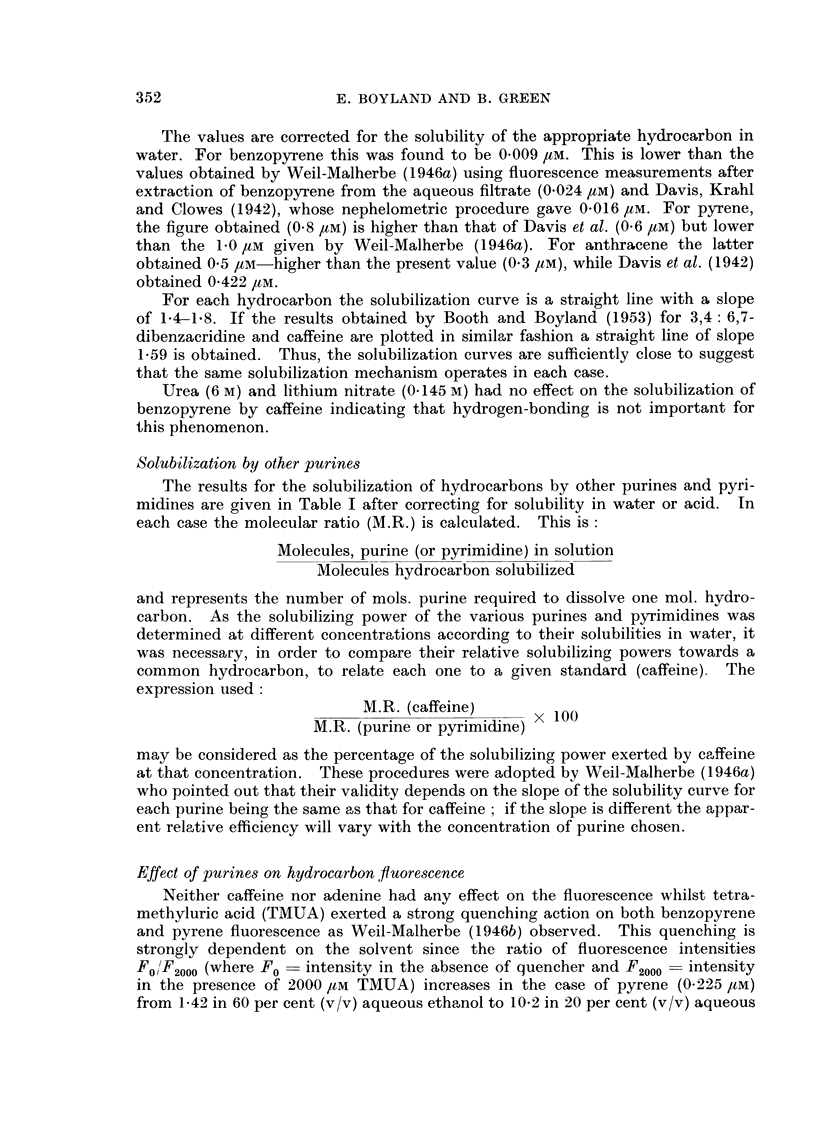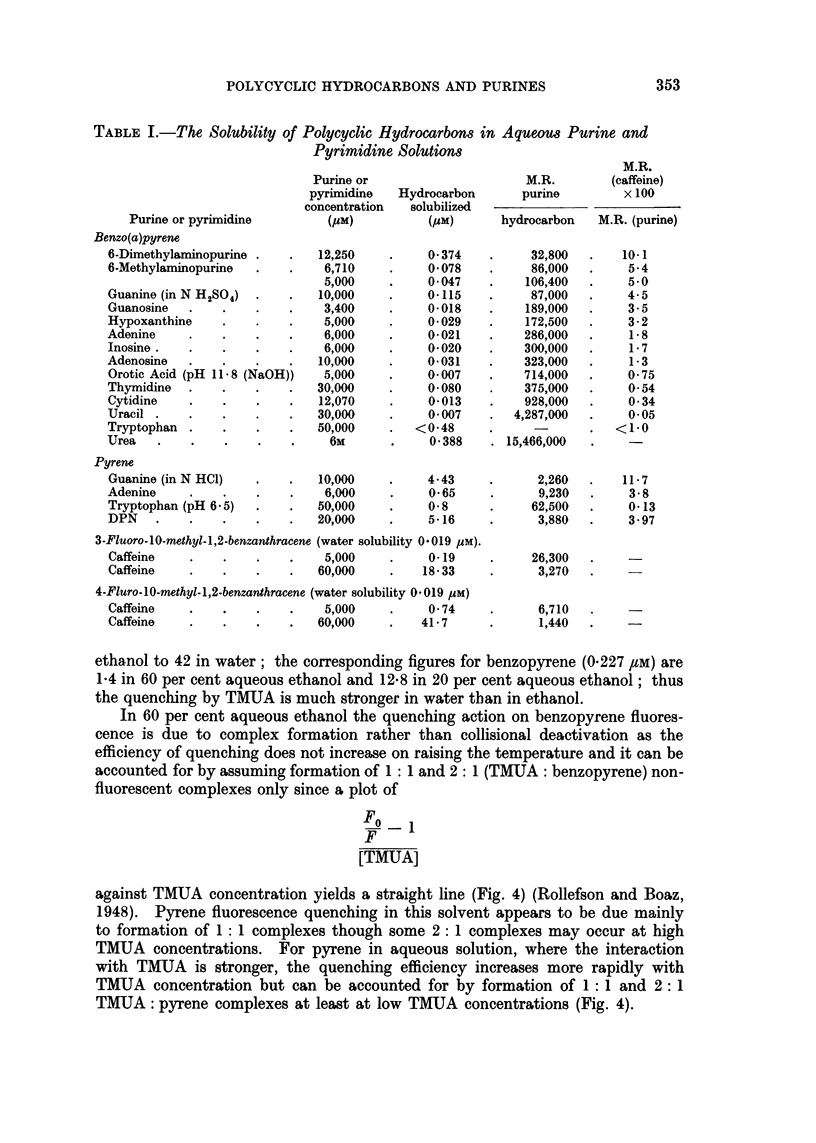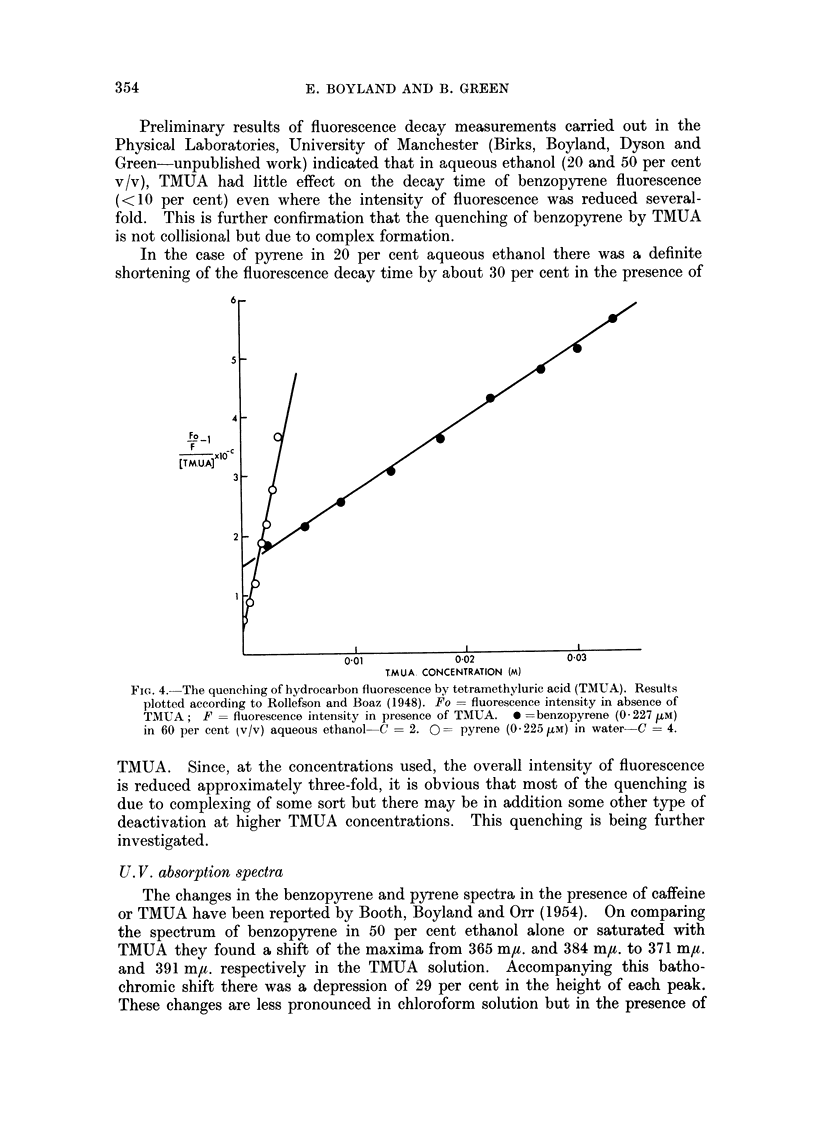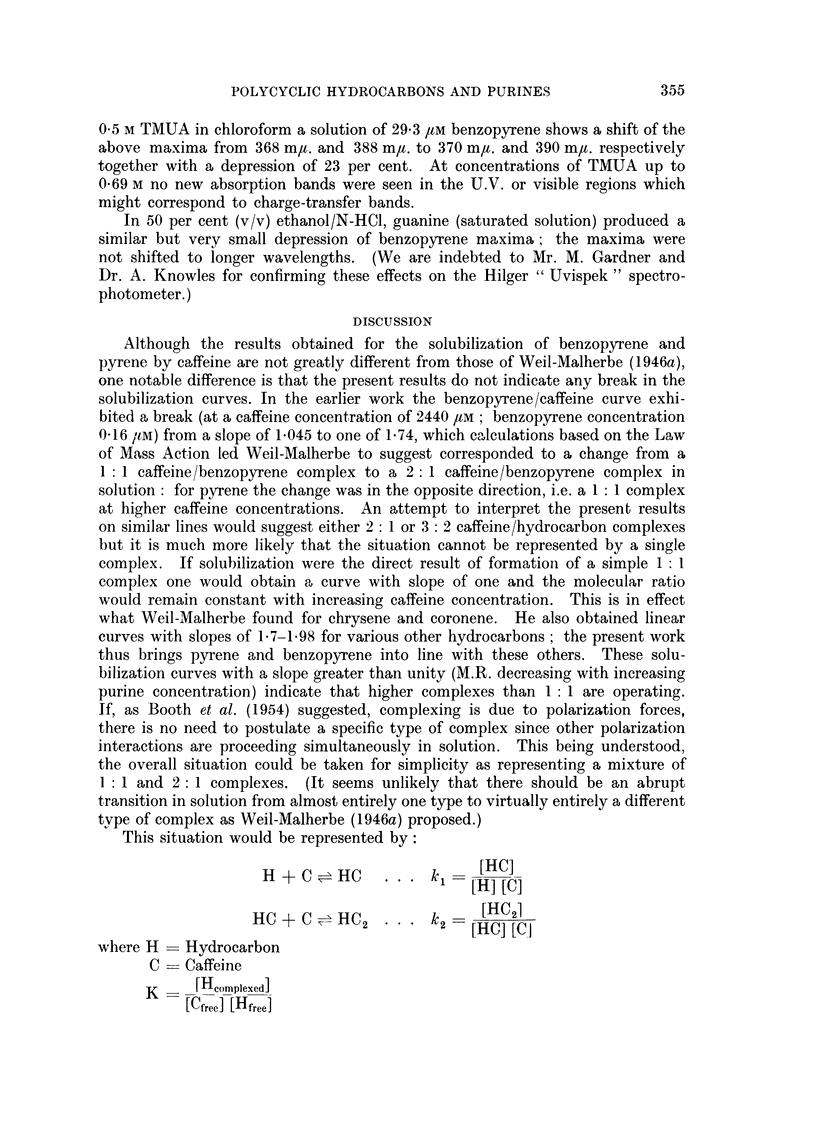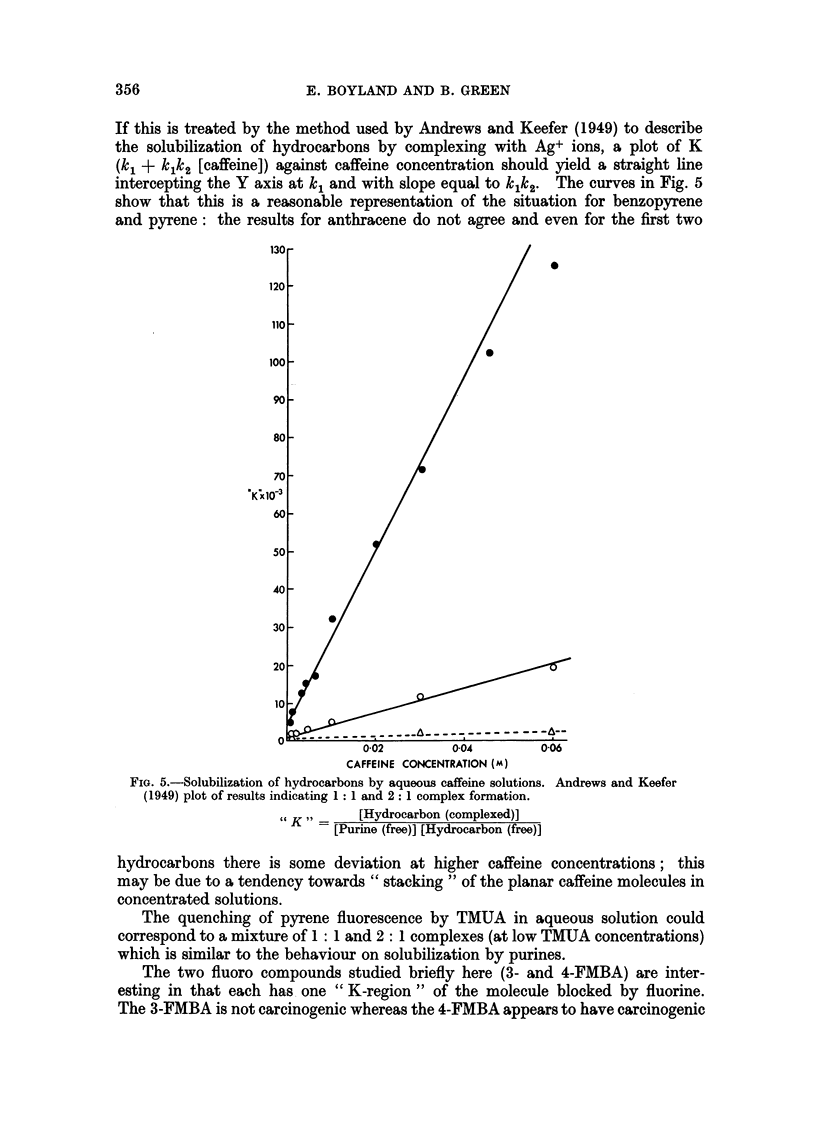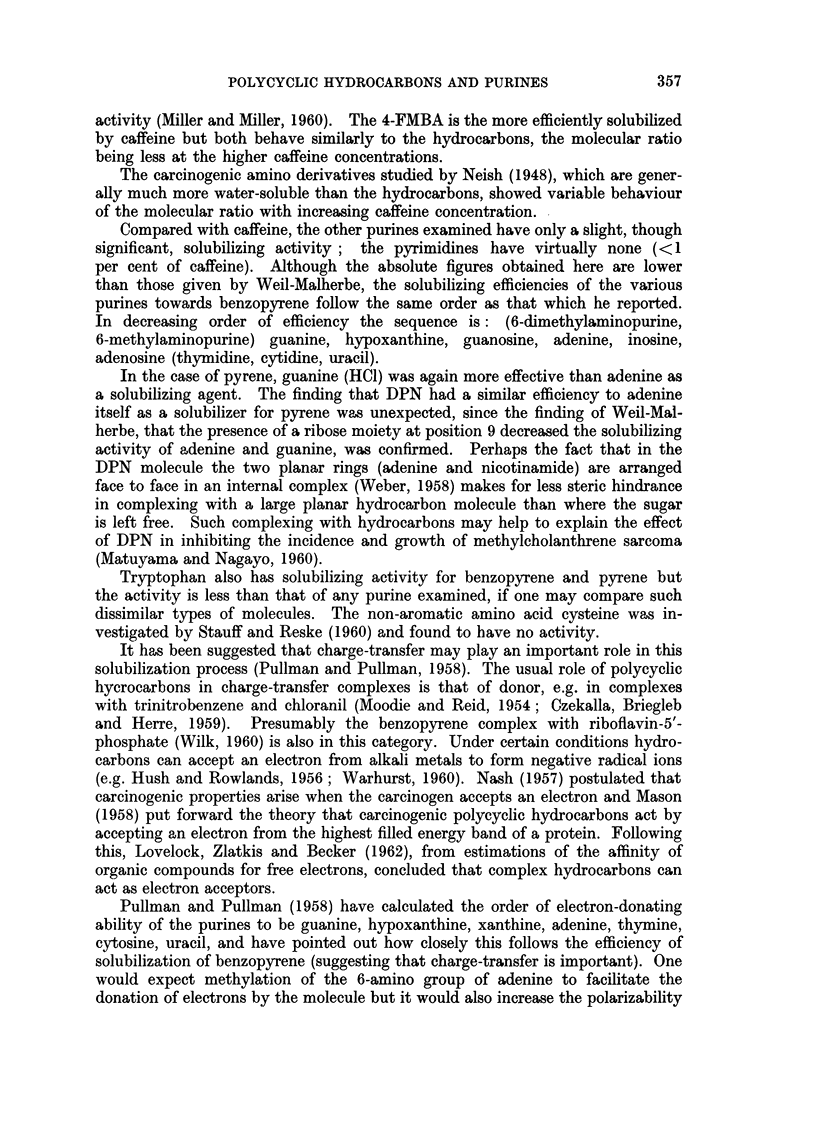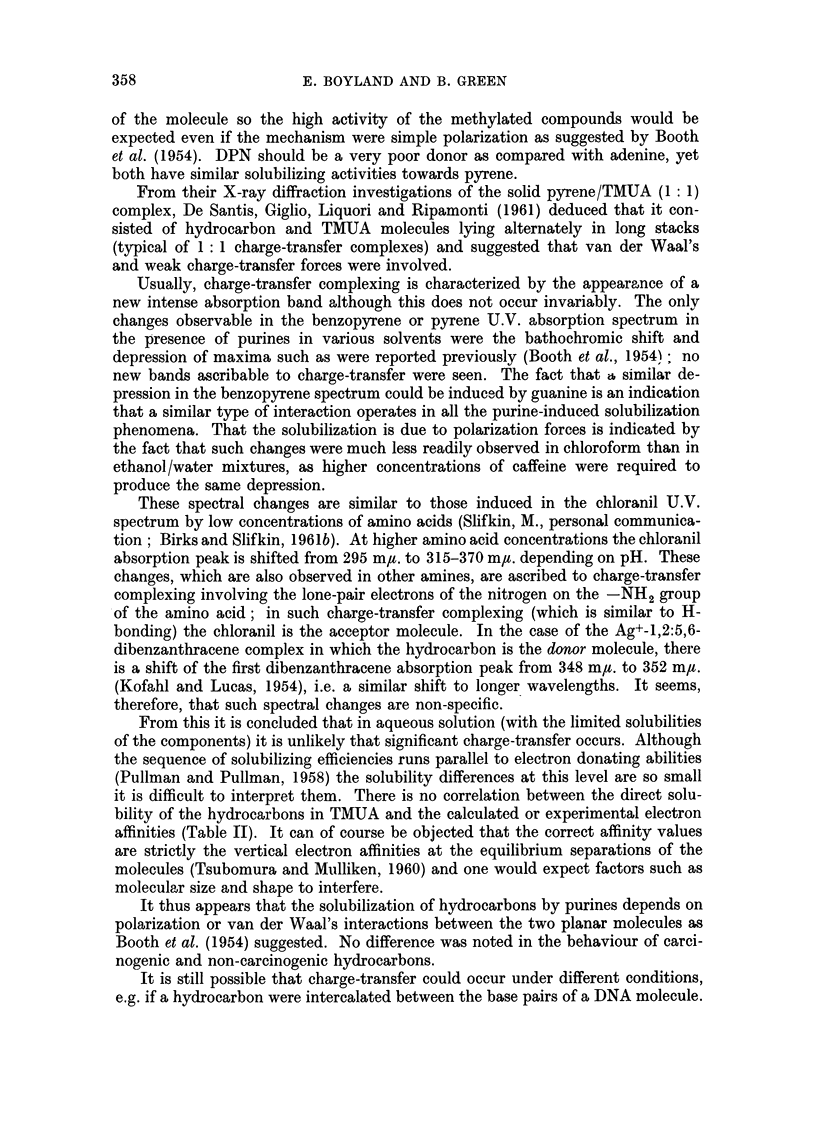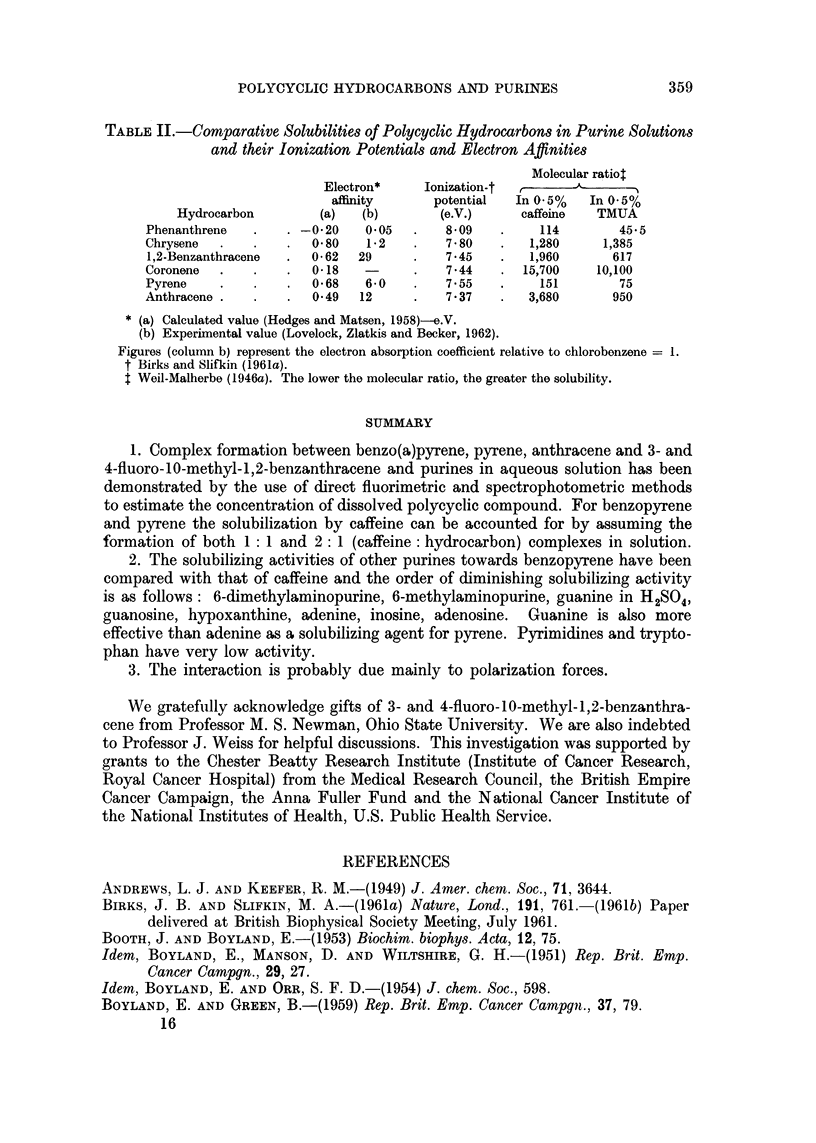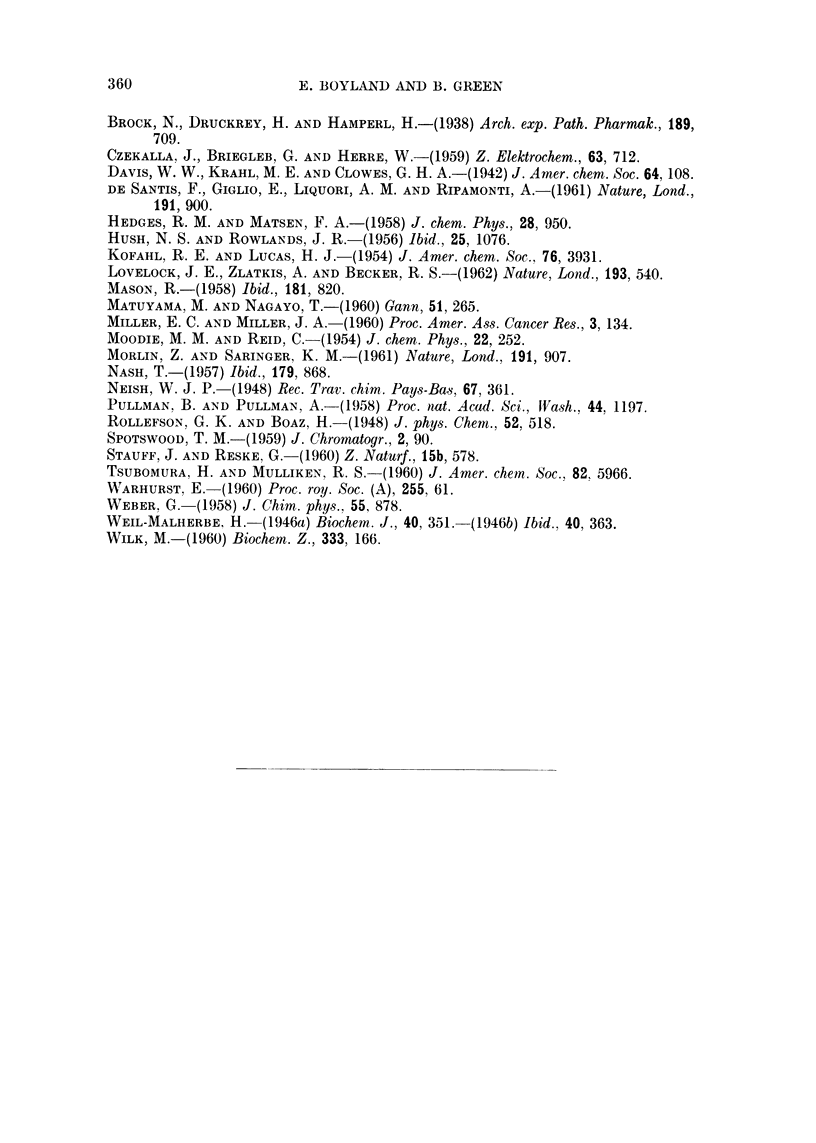# The Interaction of Polycyclic Hydrocarbons and Purines

**DOI:** 10.1038/bjc.1962.40

**Published:** 1962-06

**Authors:** E. Boyland, B. Green


### THE INTERACTION OF POLYCYCLIC HYDROCARBONS AND PURINES

E. BOYLAND AND B. GREEN

*From the Chester Beatty Research Institute, Fulham Road, London, S.W.3*

Received for publication March 8, 1962

THE reaction of purines with polycyclic hydrocarbons was first revealed by the demonstration of the solubilizing effect which aqueous solutions of caffeine, theophylline, theobromine and xanthine exerted on benzo(a)-pyrene (3,4-benzopyrene) by Brock, Druckrey and Hamperl (1938). Weil-Malherbe (1946*a*) made detailed quantitative investigations which showed that a wide range of purines exerted this solubilizing action towards many different polycyclic hydrocarbons. Other carcinogenic compounds shown to be brought into solution by purines include certain carcinogenic aromatic amines (Neish, 1948) and various dibenzocarbazoles and dibenzacridines (Booth and Boyland, 1953).

The solubilities of polycyclic hydrocarbons in most aqueous purine solutions, though greater than in water, are still small and hence difficult to estimate directly. Since many of these hydrocarbons are highly fluorescent, the availability of the Aminco-Bowman spectrophotofluorimeter provided a simple and sensitive method for the direct study of very dilute solutions of polycyclic hydrocarbons. The interaction of benzopyrene and other polycyclic hydrocarbons with various purines has been re-investigated with the aid of this instrument.

Previous work in this laboratory has shown that nucleic acids will also solubilize benzopyrene (Booth, Boyland, Manson and Wiltshire, 1951) and certain dibenzocarbazoles and dibenzacridines (Booth and Boyland, 1953). It was hoped to obtain some insight into possible mechanisms of interaction of the polycyclic hydrocarbons with nucleic acids from a study of the purines and pyrimidines themselves. The direct studies of hydrocarbon-nucleic acid interaction will be described in a succeeding paper.

### EXPERIMENTAL

### *Materials*

Benzopyrene was a commercial sample (Roche), recrystallized from benzene. Its purity was checked by the U.V. absorption spectrum after column chromatography and by chromatography on acetylated paper (Spotswood, 1959)). Pyrene and anthracene were commercial products recrystallized several times, the final recrystallizations being from ethanol. 3-Fluoro-10-methyl-1,2-benzanthracene (3 FMBA) and 4-fluoro-10-methyl-1,2-benzanthracene (4 FMBA) were supplied by Professor M. S. Newman. All these compounds were examined by chromatography on partially acetylated paper (Schleicher & Schull 2043b 21/ac): each gave a single spot except for 4 FMBA, which showed a trace of a second faster-running fluorescent component. Because of the small amount of the sample and apparent very low level of impurity, no attempt was made to purify the 4 FMBA further.

Purines and pyrimidines were all commercial products checked for purity by paper chromatography. The caffeine used in the fluorescent studies was a sample purchased from Hopkin and Williams and found to have a very low fluorescence. It proved extremely difficult to free commercial caffeine from trace fluorescent impurities ; column chromatography and recrystallization led to only slight improvement. Gradient sublimation at low pressure proved to be the best method but this is a tedious procedure where large quantities are required. The low-fluorescent commercial sample was used without further purification.

Glass-distilled water was used throughout.

*Methods*

A Unicam S.P. 500 spectrophotometer was used for measurement of U.V. absorption spectra.

Fluorescence measurements were carried out in an Aminco-Bowman spectrophotofluorimeter ; a Moseley Autograf Model 3 XY recorder was attached to the instrument when spectra were to be recorded. All the spectra are uncorrected and therefore depend on the individual instrument characteristics (variation of intensity of exciting light with wave-length, photo-multiplier response etc.).

### *Determination of Solubilities of Polyclic Compounds*

*Benzopyrene*

A suspension of benzopyrene in water was prepared according to Booth and Boyland (1953). Small volumes (0·1–0·2 ml.) of this were shaken with 100–200 ml. water or purine solution at room temperature ($\sim$22°) in a flask covered with dark paper to prevent photo-oxidation. At first the solutions were shaken for 2–3 hours, left to stand overnight, then shaken for a further 2–3 hours, but in all later work overnight shaking (16 hours) was used since slow solubilization was found to occur over this time. With longer shaking the slope of the caffeine/benzopyrene solubilization curve rose from 1·55 to 1·67. In cases where large solubilities were expected (e.g. where concentrated caffeine solutions were used) solid benzopyrene was also added. After shaking, the solutions were examined in U.V. light to ensure excess solid hydrocarbon was present.

*Estimation of benzopyrene concentration*

Where the concentration of dissolved benzopyrene was sufficiently high, it was estimated simply by measuring the U.V. absorption spectrum of the aqueous solution after removing the residual particles by filtration or centrifugation. The extinction at 391 m$\mu$. (i.e. well above the region where purines absorb) was compared with that of standard solutions of benzopyrene in the same purine solution.

With more dilute benzopyrene solutions (concentrations as low as 2 $\mu$g./l.) methods involving filtration or centrifugation sometimes gave inconsistent results due to the adsorption of the hydrocarbon from solution and the elution of fluorescent impurities from filter paper. The benzopyrene concentration was therefore estimated directly on the solution after shaking, as described by Boyland and Green (1959). The solution was allowed to stand for a few minutes to allow most of the solid particles to settle and 1–2 ml. of the supernatant was withdrawn and used for fluorescence measurements. Dissolved benzopyrene, in the Aminco-Bowman spectrophotofluorimeter, exhibits fluorescence peaks at 410 m$\mu$. and 430

mμ. and activation peaks at 295, 365 and 385 mμ. The solid benzopyrene shows a weaker broad fluorescence band with a maximum at about 490 mμ. and the main activation peak at 410 mμ. Thus, unless there is a very large excess present, the solid benzopyrene does not interfere with the fluorescence of the dissolved hydrocarbon ; this was confirmed by adding benzopyrene suspension to standard benzopyrene solutions in several different purines and measuring the fluorescence before and after the addition. For estimations, the activation maxima at 295 mμ. and 365 mμ. and fluorescence maximum at 410 mμ. were usually chosen. The fluorescence intensities were compared each time with standard solutions prepared by adding a known quantity of benzopyrene in acetone to solutions of the same concentration of the purine or pyrimidine as the unknown. The accuracy of the method depends on all the added hydrocarbon going into solution without precipitation but the concentrations are sufficiently low (below the solubility level) to render such precipitation unlikely and results from one set of standards to another were usually comparable. The concentrations of acetone used ($<0{\cdot}2$ per cent) did not significantly affect the fluorescence.

Oxygen is known to quench the fluorescence of polycyclic hydrocarbons in many solvents but the effect in aqueous solution is small due to the low solubility of oxygen in water (Weil-Malherbe, 1946*b* ; Stauff and Reske, 1960).

Experiments where the fluorescence measurements were carried out in a nitrogen atmosphere indicated that oxygen did not significantly affect the results. Similar methods have been used by Stauff and Reske (1960) and Wilk (1960).

*Other hydrocarbons and their derivatives*

Similar methods were used for pyrene and 4-FMBA. The fluorescence peaks of both these compounds in the solid state are at much longer wavelengths than the molecular solution fluorescence and hence small amounts of solid do not interfere with the estimations.

In the case of 3-FMBA and anthracene the fluorescence spectra of the solids are close to those of the dissolved compounds and hence could interfere. This is only important where the amount in true solution is very low and the fluorescence contribution from the solid is significant. The excess solid, after shaking, was removed by centrifugation (1 hour at 3300 r.p.m.)

It was noticed that occasionally the fluorescence of the standards decreased if left to stand overnight in open flasks. This may be due to the ozone effect noted by Morlin and Saringer (1961). It was most marked in the case of anthracene but if solutions were shaken in a dark sealed flask overnight the fluorescence was almost identical with freshly prepared standards. In each case freshly prepared standards were used.

Determinations were carried out in duplicate but there were variations in the results in the case of anthracene at low caffeine concentrations : in these cases the experiment was repeated until three sets of results agreed and the mean of these was taken.

### RESULTS

*Time course of solubilization*

To illustrate the method of estimation Fig. 1 shows the recordings of the fluorescence spectra of a solution of 2000 μM caffeine shaken with benzopyrene suspension ; the activation wavelength was 365 mμ. The fluorescence was measured immediately without allowing the particles to settle so that there is considerable " solid " benzopyrene fluorescence present, but the typical " dissolved " benzopyrene peaks are shown clearly. Note that both 410 mμ. and 430 mμ. peaks are shown—contrary to the report of Stauff and Reske (1960), who observed only one broad peak in caffeine solution as compared with ethanolic solution. Certainly in these aqueous solutions the 430 mμ. peak has characteristically only about two-thirds the intensity of the 410 mμ. peak but whether both peaks are detected depends on the resolution of the instrument used. The spectrum of benzopyrene in ethanol has been recorded for comparison in Fig. 1. As well as the

FIG. 1.—Fluorescence spectra of 0·002 M aqueous caffeine solution shaken with solid benzopyrene. Activation wavelength 365 mμ. Curve 1, shaken 30 seconds ; Curve 3, shaken 30 minutes ; Curve 2, benzopyrene in ethanol solution for comparison.

broadening of the bands there is also a shift of the spectrum to longer wavelengths in aqueous, as compared with ethanolic, solution.

In Fig. 2 the intensity of the 410 mμ. fluorescence peak is plotted against time of shaking. As this intensity is proportional to the concentration of benzopyrene in solution, it is clear that there is a rapid solution of hydrocarbon during the first 30 minutes followed by a further slow increase, but solution is almost complete after 3 hours, there being only a very slow increase after this ; the 16 hour shaking period has been taken as being sufficiently close to equilibrium for these experiments.

*Solubilization of hydrocarbons by caffeine*

Fig. 3 shows the results obtained for the solubilization of benzopyrene, pyrene, and anthracene by caffeine over the concentration range 250–60,000 μM plotted on double-logarithmic paper following the procedure of Weil-Malherbe (1946*a*).

FIG. 2.—Increase in fluorescence intensity at 410 mμ. (corresponding to dissolved benzopyrene) of samples of 0·002 M aqueous caffeine solution removed after shaking with solid benzopyrene for various times. Activating wavelength 365 mμ.

FIG. 3.—The solubilization of polycyclic hydrocarbons by aqueous caffeine solutions. ● = benzopyrene ; ○ = pyrene ; △ = anthracene.

The values are corrected for the solubility of the appropriate hydrocarbon in water. For benzopyrene this was found to be 0·009 $\mu$M. This is lower than the values obtained by Weil-Malherbe (1946*a*) using fluorescence measurements after extraction of benzopyrene from the aqueous filtrate (0·024 $\mu$M) and Davis, Krahl and Clowes (1942), whose nephelometric procedure gave 0·016 $\mu$M. For pyrene, the figure obtained (0·8 $\mu$M) is higher than that of Davis *et al.* (0·6 $\mu$M) but lower than the 1·0 $\mu$M given by Weil-Malherbe (1946*a*). For anthracene the latter obtained 0·5 $\mu$M—higher than the present value (0·3 $\mu$M), while Davis *et al.* (1942) obtained 0·422 $\mu$M.

For each hydrocarbon the solubilization curve is a straight line with a slope of 1·4–1·8. If the results obtained by Booth and Boyland (1953) for 3,4 : 6,7-dibenzacridine and caffeine are plotted in similar fashion a straight line of slope 1·59 is obtained. Thus, the solubilization curves are sufficiently close to suggest that the same solubilization mechanism operates in each case.

Urea (6 M) and lithium nitrate (0·145 M) had no effect on the solubilization of benzopyrene by caffeine indicating that hydrogen-bonding is not important for this phenomenon.

*Solubilization by other purines*

The results for the solubilization of hydrocarbons by other purines and pyrimidines are given in Table I after correcting for solubility in water or acid. In each case the molecular ratio (M.R.) is calculated. This is :

$$\frac{\text{Molecules, purine (or pyrimidine) in solution}}{\text{Molecules hydrocarbon solubilized}}$$

and represents the number of mols. purine required to dissolve one mol. hydrocarbon. As the solubilizing power of the various purines and pyrimidines was determined at different concentrations according to their solubilities in water, it was necessary, in order to compare their relative solubilizing powers towards a common hydrocarbon, to relate each one to a given standard (caffeine). The expression used :

$$\frac{\text{M.R. (caffeine)}}{\text{M.R. (purine or pyrimidine)}} \times 100$$

may be considered as the percentage of the solubilizing power exerted by caffeine at that concentration. These procedures were adopted by Weil-Malherbe (1946*a*) who pointed out that their validity depends on the slope of the solubility curve for each purine being the same as that for caffeine ; if the slope is different the apparent relative efficiency will vary with the concentration of purine chosen.

*Effect of purines on hydrocarbon fluorescence*

Neither caffeine nor adenine had any effect on the fluorescence whilst tetramethyluric acid (TMUA) exerted a strong quenching action on both benzopyrene and pyrene fluorescence as Weil-Malherbe (1946*b*) observed. This quenching is strongly dependent on the solvent since the ratio of fluorescence intensities $F_0/F_{2000}$ (where $F_0$ = intensity in the absence of quencher and $F_{2000}$ = intensity in the presence of 2000 $\mu$M TMUA) increases in the case of pyrene (0·225 $\mu$M) from 1·42 in 60 per cent (v/v) aqueous ethanol to 10·2 in 20 per cent (v/v) aqueous

TABLE I.—*The Solubility of Polycyclic Hydrocarbons in Aqueous Purine and Pyrimidine Solutions*

| Purine or pyrimidine | Purine or pyrimidine concentration (μM) | Hydrocarbon solubilized (μM) | M.R. purine / hydrocarbon | M.R. (caffeine) ×100 / M.R. (purine) |
|---|---|---|---|---|
| *Benzo(a)pyrene* | | | | |
| 6-Dimethylaminopurine | 12,250 | 0·374 | 32,800 | 10·1 |
| 6-Methylaminopurine | 6,710 | 0·078 | 86,000 | 5·4 |
| | 5,000 | 0·047 | 106,400 | 5·0 |
| Guanine (in N $H_2SO_4$) | 10,000 | 0·115 | 87,000 | 4·5 |
| Guanosine | 3,400 | 0·018 | 189,000 | 3·5 |
| Hypoxanthine | 5,000 | 0·029 | 172,500 | 3·2 |
| Adenine | 6,000 | 0·021 | 286,000 | 1·8 |
| Inosine | 6,000 | 0·020 | 300,000 | 1·7 |
| Adenosine | 10,000 | 0·031 | 323,000 | 1·3 |
| Orotic Acid (pH 11·8 (NaOH)) | 5,000 | 0·007 | 714,000 | 0·75 |
| Thymidine | 30,000 | 0·080 | 375,000 | 0·54 |
| Cytidine | 12,070 | 0·013 | 928,000 | 0·34 |
| Uracil | 30,000 | 0·007 | 4,287,000 | 0·05 |
| Tryptophan | 50,000 | <0·48 | — | <1·0 |
| Urea | 6M | 0·388 | 15,466,000 | — |
| *Pyrene* | | | | |
| Guanine (in N HCl) | 10,000 | 4·43 | 2,260 | 11·7 |
| Adenine | 6,000 | 0·65 | 9,230 | 3·8 |
| Tryptophan (pH 6·5) | 50,000 | 0·8 | 62,500 | 0·13 |
| DPN | 20,000 | 5·16 | 3,880 | 3·97 |
| 3-*Fluoro*-10-*methyl*-1,2-*benzanthracene* (water solubility 0·019 μM). | | | | |
| Caffeine | 5,000 | 0·19 | 26,300 | — |
| Caffeine | 60,000 | 18·33 | 3,270 | — |
| 4-*Fluro*-10-*methyl*-1,2-*benzanthracene* (water solubility 0·019 μM) | | | | |
| Caffeine | 5,000 | 0·74 | 6,710 | — |
| Caffeine | 60,000 | 41·7 | 1,440 | — |

ethanol to 42 in water ; the corresponding figures for benzopyrene (0·227 μM) are 1·4 in 60 per cent aqueous ethanol and 12·8 in 20 per cent aqueous ethanol ; thus the quenching by TMUA is much stronger in water than in ethanol.

In 60 per cent aqueous ethanol the quenching action on benzopyrene fluorescence is due to complex formation rather than collisional deactivation as the efficiency of quenching does not increase on raising the temperature and it can be accounted for by assuming formation of 1 : 1 and 2 : 1 (TMUA : benzopyrene) non-fluorescent complexes only since a plot of

$$\frac{\frac{F_0}{F} - 1}{[TMUA]}$$

against TMUA concentration yields a straight line (Fig. 4) (Rollefson and Boaz, 1948). Pyrene fluorescence quenching in this solvent appears to be due mainly to formation of 1 : 1 complexes though some 2 : 1 complexes may occur at high TMUA concentrations. For pyrene in aqueous solution, where the interaction with TMUA is stronger, the quenching efficiency increases more rapidly with TMUA concentration but can be accounted for by formation of 1 : 1 and 2 : 1 TMUA : pyrene complexes at least at low TMUA concentrations (Fig. 4).

Preliminary results of fluorescence decay measurements carried out in the Physical Laboratories, University of Manchester (Birks, Boyland, Dyson and Green—unpublished work) indicated that in aqueous ethanol (20 and 50 per cent v/v), TMUA had little effect on the decay time of benzopyrene fluorescence ($<10$ per cent) even where the intensity of fluorescence was reduced several-fold. This is further confirmation that the quenching of benzopyrene by TMUA is not collisional but due to complex formation.

In the case of pyrene in 20 per cent aqueous ethanol there was a definite shortening of the fluorescence decay time by about 30 per cent in the presence of

FIG. 4.—The quenching of hydrocarbon fluorescence by tetramethyluric acid (TMUA). Results plotted according to Rollefson and Boaz (1948). *Fo* = fluorescence intensity in absence of TMUA ; *F* = fluorescence intensity in presence of TMUA. ● = benzopyrene (0·227 μM) in 60 per cent (v/v) aqueous ethanol—$C = 2$. ○ = pyrene (0·225 μM) in water—$C = 4$.

TMUA. Since, at the concentrations used, the overall intensity of fluorescence is reduced approximately three-fold, it is obvious that most of the quenching is due to complexing of some sort but there may be in addition some other type of deactivation at higher TMUA concentrations. This quenching is being further investigated.

*U.V. absorption spectra*

The changes in the benzopyrene and pyrene spectra in the presence of caffeine or TMUA have been reported by Booth, Boyland and Orr (1954). On comparing the spectrum of benzopyrene in 50 per cent ethanol alone or saturated with TMUA they found a shift of the maxima from 365 mμ. and 384 mμ. to 371 mμ. and 391 mμ. respectively in the TMUA solution. Accompanying this bathochromic shift there was a depression of 29 per cent in the height of each peak. These changes are less pronounced in chloroform solution but in the presence of

0·5 M TMUA in chloroform a solution of 29·3 μM benzopyrene shows a shift of the above maxima from 368 mμ. and 388 mμ. to 370 mμ. and 390 mμ. respectively together with a depression of 23 per cent. At concentrations of TMUA up to 0·69 M no new absorption bands were seen in the U.V. or visible regions which might correspond to charge-transfer bands.

In 50 per cent (v/v) ethanol/N-HCl, guanine (saturated solution) produced a similar but very small depression of benzopyrene maxima; the maxima were not shifted to longer wavelengths. (We are indebted to Mr. M. Gardner and Dr. A. Knowles for confirming these effects on the Hilger "Uvispek" spectrophotometer.)

### DISCUSSION

Although the results obtained for the solubilization of benzopyrene and pyrene by caffeine are not greatly different from those of Weil-Malherbe (1946*a*), one notable difference is that the present results do not indicate any break in the solubilization curves. In the earlier work the benzopyrene/caffeine curve exhibited a break (at a caffeine concentration of 2440 μM; benzopyrene concentration 0·16 μM) from a slope of 1·045 to one of 1·74, which calculations based on the Law of Mass Action led Weil-Malherbe to suggest corresponded to a change from a 1 : 1 caffeine/benzopyrene complex to a 2 : 1 caffeine/benzopyrene complex in solution: for pyrene the change was in the opposite direction, i.e. a 1 : 1 complex at higher caffeine concentrations. An attempt to interpret the present results on similar lines would suggest either 2 : 1 or 3 : 2 caffeine/hydrocarbon complexes but it is much more likely that the situation cannot be represented by a single complex. If solubilization were the direct result of formation of a simple 1 : 1 complex one would obtain a curve with slope of one and the molecular ratio would remain constant with increasing caffeine concentration. This is in effect what Weil-Malherbe found for chrysene and coronene. He also obtained linear curves with slopes of 1·7–1·98 for various other hydrocarbons; the present work thus brings pyrene and benzopyrene into line with these others. These solubilization curves with a slope greater than unity (M.R. decreasing with increasing purine concentration) indicate that higher complexes than 1 : 1 are operating. If, as Booth *et al.* (1954) suggested, complexing is due to polarization forces, there is no need to postulate a specific type of complex since other polarization interactions are proceeding simultaneously in solution. This being understood, the overall situation could be taken for simplicity as representing a mixture of 1 : 1 and 2 : 1 complexes. (It seems unlikely that there should be an abrupt transition in solution from almost entirely one type to virtually entirely a different type of complex as Weil-Malherbe (1946*a*) proposed.)

This situation would be represented by:

$$H + C \rightleftharpoons HC \quad \ldots \quad k_1 = \frac{[HC]}{[H]\,[C]}$$

$$HC + C \rightleftharpoons HC_2 \quad \ldots \quad k_2 = \frac{[HC_2]}{[HC]\,[C]}$$

where H = Hydrocarbon
C = Caffeine

$$K = \frac{[H_{complexed}]}{[C_{free}]\,[H_{free}]}$$

If this is treated by the method used by Andrews and Keefer (1949) to describe the solubilization of hydrocarbons by complexing with $Ag^+$ ions, a plot of K ($k_1 + k_1k_2$ [caffeine]) against caffeine concentration should yield a straight line intercepting the Y axis at $k_1$ and with slope equal to $k_1k_2$. The curves in Fig. 5 show that this is a reasonable representation of the situation for benzopyrene and pyrene: the results for anthracene do not agree and even for the first two

FIG. 5.—Solubilization of hydrocarbons by aqueous caffeine solutions. Andrews and Keefer (1949) plot of results indicating 1 : 1 and 2 : 1 complex formation.

$$\text{“}K\text{”} = \frac{[\text{Hydrocarbon (complexed)}]}{[\text{Purine (free)}]\ [\text{Hydrocarbon (free)}]}$$

hydrocarbons there is some deviation at higher caffeine concentrations; this may be due to a tendency towards " stacking " of the planar caffeine molecules in concentrated solutions.

The quenching of pyrene fluorescence by TMUA in aqueous solution could correspond to a mixture of 1 : 1 and 2 : 1 complexes (at low TMUA concentrations) which is similar to the behaviour on solubilization by purines.

The two fluoro compounds studied briefly here (3- and 4-FMBA) are interesting in that each has one " K-region " of the molecule blocked by fluorine. The 3-FMBA is not carcinogenic whereas the 4-FMBA appears to have carcinogenic activity (Miller and Miller, 1960). The 4-FMBA is the more efficiently solubilized by caffeine but both behave similarly to the hydrocarbons, the molecular ratio being less at the higher caffeine concentrations.

The carcinogenic amino derivatives studied by Neish (1948), which are generally much more water-soluble than the hydrocarbons, showed variable behaviour of the molecular ratio with increasing caffeine concentration.

Compared with caffeine, the other purines examined have only a slight, though significant, solubilizing activity ; the pyrimidines have virtually none ($<1$ per cent of caffeine). Although the absolute figures obtained here are lower than those given by Weil-Malherbe, the solubilizing efficiencies of the various purines towards benzopyrene follow the same order as that which he reported. In decreasing order of efficiency the sequence is : (6-dimethylaminopurine, 6-methylaminopurine) guanine, hypoxanthine, guanosine, adenine, inosine, adenosine (thymidine, cytidine, uracil).

In the case of pyrene, guanine (HCl) was again more effective than adenine as a solubilizing agent. The finding that DPN had a similar efficiency to adenine itself as a solubilizer for pyrene was unexpected, since the finding of Weil-Malherbe, that the presence of a ribose moiety at position 9 decreased the solubilizing activity of adenine and guanine, was confirmed. Perhaps the fact that in the DPN molecule the two planar rings (adenine and nicotinamide) are arranged face to face in an internal complex (Weber, 1958) makes for less steric hindrance in complexing with a large planar hydrocarbon molecule than where the sugar is left free. Such complexing with hydrocarbons may help to explain the effect of DPN in inhibiting the incidence and growth of methylcholanthrene sarcoma (Matuyama and Nagayo, 1960).

Tryptophan also has solubilizing activity for benzopyrene and pyrene but the activity is less than that of any purine examined, if one may compare such dissimilar types of molecules. The non-aromatic amino acid cysteine was investigated by Stauff and Reske (1960) and found to have no activity.

It has been suggested that charge-transfer may play an important role in this solubilization process (Pullman and Pullman, 1958). The usual role of polycyclic hycrocarbons in charge-transfer complexes is that of donor, e.g. in complexes with trinitrobenzene and chloranil (Moodie and Reid, 1954 ; Czekalla, Briegleb and Herre, 1959). Presumably the benzopyrene complex with riboflavin-5′-phosphate (Wilk, 1960) is also in this category. Under certain conditions hydrocarbons can accept an electron from alkali metals to form negative radical ions (e.g. Hush and Rowlands, 1956 ; Warhurst, 1960). Nash (1957) postulated that carcinogenic properties arise when the carcinogen accepts an electron and Mason (1958) put forward the theory that carcinogenic polycyclic hydrocarbons act by accepting an electron from the highest filled energy band of a protein. Following this, Lovelock, Zlatkis and Becker (1962), from estimations of the affinity of organic compounds for free electrons, concluded that complex hydrocarbons can act as electron acceptors.

Pullman and Pullman (1958) have calculated the order of electron-donating ability of the purines to be guanine, hypoxanthine, xanthine, adenine, thymine, cytosine, uracil, and have pointed out how closely this follows the efficiency of solubilization of benzopyrene (suggesting that charge-transfer is important). One would expect methylation of the 6-amino group of adenine to facilitate the donation of electrons by the molecule but it would also increase the polarizability of the molecule so the high activity of the methylated compounds would be expected even if the mechanism were simple polarization as suggested by Booth *et al.* (1954). DPN should be a very poor donor as compared with adenine, yet both have similar solubilizing activities towards pyrene.

From their X-ray diffraction investigations of the solid pyrene/TMUA (1 : 1) complex, De Santis, Giglio, Liquori and Ripamonti (1961) deduced that it consisted of hydrocarbon and TMUA molecules lying alternately in long stacks (typical of 1 : 1 charge-transfer complexes) and suggested that van der Waal's and weak charge-transfer forces were involved.

Usually, charge-transfer complexing is characterized by the appearance of a new intense absorption band although this does not occur invariably. The only changes observable in the benzopyrene or pyrene U.V. absorption spectrum in the presence of purines in various solvents were the bathochromic shift and depression of maxima such as were reported previously (Booth *et al.*, 1954) ; no new bands ascribable to charge-transfer were seen. The fact that a similar depression in the benzopyrene spectrum could be induced by guanine is an indication that a similar type of interaction operates in all the purine-induced solubilization phenomena. That the solubilization is due to polarization forces is indicated by the fact that such changes were much less readily observed in chloroform than in ethanol/water mixtures, as higher concentrations of caffeine were required to produce the same depression.

These spectral changes are similar to those induced in the chloranil U.V. spectrum by low concentrations of amino acids (Slifkin, M., personal communication ; Birks and Slifkin, 1961*b*). At higher amino acid concentrations the chloranil absorption peak is shifted from 295 m$\mu$. to 315–370 m$\mu$. depending on pH. These changes, which are also observed in other amines, are ascribed to charge-transfer complexing involving the lone-pair electrons of the nitrogen on the $-NH_2$ group of the amino acid ; in such charge-transfer complexing (which is similar to H-bonding) the chloranil is the acceptor molecule. In the case of the $Ag^+$-1,2:5,6-dibenzanthracene complex in which the hydrocarbon is the *donor* molecule, there is a shift of the first dibenzanthracene absorption peak from 348 m$\mu$. to 352 m$\mu$. (Kofahl and Lucas, 1954), i.e. a similar shift to longer wavelengths. It seems, therefore, that such spectral changes are non-specific.

From this it is concluded that in aqueous solution (with the limited solubilities of the components) it is unlikely that significant charge-transfer occurs. Although the sequence of solubilizing efficiencies runs parallel to electron donating abilities (Pullman and Pullman, 1958) the solubility differences at this level are so small it is difficult to interpret them. There is no correlation between the direct solubility of the hydrocarbons in TMUA and the calculated or experimental electron affinities (Table II). It can of course be objected that the correct affinity values are strictly the vertical electron affinities at the equilibrium separations of the molecules (Tsubomura and Mulliken, 1960) and one would expect factors such as molecular size and shape to interfere.

It thus appears that the solubilization of hydrocarbons by purines depends on polarization or van der Waal's interactions between the two planar molecules as Booth *et al.* (1954) suggested. No difference was noted in the behaviour of carcinogenic and non-carcinogenic hydrocarbons.

It is still possible that charge-transfer could occur under different conditions, e.g. if a hydrocarbon were intercalated between the base pairs of a DNA molecule.

TABLE II.—*Comparative Solubilities of Polycyclic Hydrocarbons in Purine Solutions and their Ionization Potentials and Electron Affinities*

| Hydrocarbon | Electron* affinity (a) | Electron* affinity (b) | Ionization-† potential (e.V.) | Molecular ratio‡ In 0·5% caffeine | Molecular ratio‡ In 0·5% TMUA |
|---|---|---|---|---|---|
| Phenanthrene | −0·20 | 0·05 | 8·09 | 114 | 45·5 |
| Chrysene | 0·80 | 1·2 | 7·80 | 1,280 | 1,385 |
| 1,2-Benzanthracene | 0·62 | 29 | 7·45 | 1,960 | 617 |
| Coronene | 0·18 | — | 7·44 | 15,700 | 10,100 |
| Pyrene | 0·68 | 6·0 | 7·55 | 151 | 75 |
| Anthracene | 0·49 | 12 | 7·37 | 3,680 | 950 |

* (a) Calculated value (Hedges and Matsen, 1958)—e.V.
(b) Experimental value (Lovelock, Zlatkis and Becker, 1962).

Figures (column b) represent the electron absorption coefficient relative to chlorobenzene = 1.
† Birks and Slifkin (1961*a*).
‡ Weil-Malherbe (1946*a*). The lower the molecular ratio, the greater the solubility.

### SUMMARY

1. Complex formation between benzo(a)pyrene, pyrene, anthracene and 3- and 4-fluoro-10-methyl-1,2-benzanthracene and purines in aqueous solution has been demonstrated by the use of direct fluorimetric and spectrophotometric methods to estimate the concentration of dissolved polycyclic compound. For benzopyrene and pyrene the solubilization by caffeine can be accounted for by assuming the formation of both 1 : 1 and 2 : 1 (caffeine : hydrocarbon) complexes in solution.

2. The solubilizing activities of other purines towards benzopyrene have been compared with that of caffeine and the order of diminishing solubilizing activity is as follows : 6-dimethylaminopurine, 6-methylaminopurine, guanine in $H_2SO_4$, guanosine, hypoxanthine, adenine, inosine, adenosine. Guanine is also more effective than adenine as a solubilizing agent for pyrene. Pyrimidines and tryptophan have very low activity.

3. The interaction is probably due mainly to polarization forces.

We gratefully acknowledge gifts of 3- and 4-fluoro-10-methyl-1,2-benzanthracene from Professor M. S. Newman, Ohio State University. We are also indebted to Professor J. Weiss for helpful discussions. This investigation was supported by grants to the Chester Beatty Research Institute (Institute of Cancer Research, Royal Cancer Hospital) from the Medical Research Council, the British Empire Cancer Campaign, the Anna Fuller Fund and the National Cancer Institute of the National Institutes of Health, U.S. Public Health Service.

### REFERENCES

ANDREWS, L. J. AND KEEFER, R. M.—(1949) *J. Amer. chem. Soc.*, **71**, 3644.

BIRKS, J. B. AND SLIFKIN, M. A.—(1961*a*) *Nature, Lond.*, **191**, 761.—(1961*b*) Paper delivered at British Biophysical Society Meeting, July 1961.

BOOTH, J. AND BOYLAND, E.—(1953) *Biochim. biophys. Acta*, **12**, 75.

*Idem*, BOYLAND, E., MANSON, D. AND WILTSHIRE, G. H.—(1951) *Rep. Brit. Emp. Cancer Campgn.*, **29**, 27.

*Idem*, BOYLAND, E. AND ORR, S. F. D.—(1954) *J. chem. Soc.*, 598.

BOYLAND, E. AND GREEN, B.—(1959) *Rep. Brit. Emp. Cancer Campgn.*, **37**, 79.

BROCK, N., DRUCKREY, H. AND HAMPERL, H.—(1938) *Arch. exp. Path. Pharmak.*, **189**, 709.
CZEKALLA, J., BRIEGLEB, G. AND HERRE, W.—(1959) *Z. Elektrochem.*, **63**, 712.
DAVIS, W. W., KRAHL, M. E. AND CLOWES, G. H. A.—(1942) *J. Amer. chem. Soc.* **64**, 108.
DE SANTIS, F., GIGLIO, E., LIQUORI, A. M. AND RIPAMONTI, A.—(1961) *Nature, Lond.*, **191**, 900.
HEDGES, R. M. AND MATSEN, F. A.—(1958) *J. chem. Phys.*, **28**, 950.
HUSH, N. S. AND ROWLANDS, J. R.—(1956) *Ibid.*, **25**, 1076.
KOFAHL, R. E. AND LUCAS, H. J.—(1954) *J. Amer. chem. Soc.*, **76**, 3931.
LOVELOCK, J. E., ZLATKIS, A. AND BECKER, R. S.—(1962) *Nature, Lond.*, **193**, 540.
MASON, R.—(1958) *Ibid.*, **181**, 820.
MATUYAMA, M. AND NAGAYO, T.—(1960) *Gann*, **51**, 265.
MILLER, E. C. AND MILLER, J. A.—(1960) *Proc. Amer. Ass. Cancer Res.*, **3**, 134.
MOODIE, M. M. AND REID, C.—(1954) *J. chem. Phys.*, **22**, 252.
MORLIN, Z. AND SARINGER, K. M.—(1961) *Nature, Lond.*, **191**, 907.
NASH, T.—(1957) *Ibid.*, **179**, 868.
NEISH, W. J. P.—(1948) *Rec. Trav. chim. Pays-Bas*, **67**, 361.
PULLMAN, B. AND PULLMAN, A.—(1958) *Proc. nat. Acad. Sci., Wash.*, **44**, 1197.
ROLLEFSON, G. K. AND BOAZ, H.—(1948) *J. phys. Chem.*, **52**, 518.
SPOTSWOOD, T. M.—(1959) *J. Chromatogr.*, **2**, 90.
STAUFF, J. AND RESKE, G.—(1960) *Z. Naturf.*, **15b**, 578.
TSUBOMURA, H. AND MULLIKEN, R. S.—(1960) *J. Amer. chem. Soc.*, **82**, 5966.
WARHURST, E.—(1960) *Proc. roy. Soc.* (A), **255**, 61.
WEBER, G.—(1958) *J. Chim. phys.*, **55**, 878.
WEIL-MALHERBE, H.—(1946*a*) *Biochem. J.*, **40**, 351.—(1946*b*) *Ibid.*, **40**, 363.
WILK, M.—(1960) *Biochem. Z.*, **333**, 166.